# Food security and dietary intake of a cohort of South African students during COVID-19

**DOI:** 10.4102/hsag.v30i0.2711

**Published:** 2025-01-20

**Authors:** Juanita Jonker, Corinna Walsh

**Affiliations:** 1 Department of Health Sciences, Faculty of Health and Environmental Sciences, Central University of Technology, Bloemfontein, South Africa; 2 Department of Nutrition and Dietetics, Faculty of Health Sciences, University of the Free State, Bloemfontein, South Africa

**Keywords:** COVID-19, higher education, ICE environment, health sciences students, food security, diet, institutional support

## Abstract

**Background:**

Food insecurity among students was a global concern even before the coronavirus disease 2019 (COVID-19) pandemic. Food security comprises having access to sufficient, safe and nutritious food at all times. The COVID-19 containment measures negatively influenced economies, impacting citizens’ food security.

**Aim:**

This study aimed to investigate the food security of a cohort of South African students during the COVID-19 pandemic.

**Setting:**

The sample included Health Science students from a university in Central South Africa.

**Methods:**

A mixed-method study was performed using questionnaires and focus group discussions. The questionnaire and focus group discussions investigated similar areas and were supported by literature. Ethical clearance was obtained.

**Results:**

Food insecurity existed among 84% of questionnaire participants. Dietary intake changed, with mainly a decrease in sugary and salty snacks. Focus group participants indicated an increase in the intake of starchy foods and reverted to binge eating. Dietary intake changes were attributed to poor availability and limited resources, for example money and electricity. Few participants were aware of or utilised available support services.

**Conclusion:**

The isolated, confined and extreme (ICE) environment that resulted from COVID-19 negatively impacted students’ dietary intake and food security. Institutions should implement measures to support students’ intake of healthy foods during ICE events.

**Contribution:**

This study provides significant insights into the dietary intake and food security of a cohort of Health Science students during COVID-19. It highlights the need for improved institutional and government food relief interventions during future pandemics. Therefore, this study contributes to the second sustainable development goal namely: zero hunger.

## Introduction

The extreme regulations enforced to contain the coronavirus disease 2019 (COVID-19) led to isolated, confined and extreme (ICE) environments. Such regulations included social distancing, movement restrictions and temporary closure of businesses. The COVID-19 regulations substantially impacted the workings of the state and the economy. Furthermore, on an individual level, it impacted citizens’ lives with sudden lifestyle changes, increased anxiety and decreased income (Di Renzo et al. 2020). In developing countries such as South Africa, the pandemic exacerbated an already burdened country where meeting the basic needs of the underprivileged population was already a challenge (Workie et al. 2020). Moreover, early research during the pandemic indicated that the COVID-19 containment measures had a negative impact on economies globally, which in turn impacted food security (Anelich et al. 2020). International studies indicated an increase in food insecurity and dietary intake changes, especially in rural and low-income households, during early COVID-19 (Anelich et al. 2020).

Food security is present ‘when all people, at all times, have physical, social and economic access to sufficient, safe and nutritious food which meets their dietary needs and food preferences for an active and healthy life’ (HLPE 2020:online). According to the Food and Agriculture Organization (FAO), food security includes six dimensions that can be measured by using several metrics, such as self-reporting surveys (Coates et al. 2007). Different metrics measure different aspects of food security. Therefore, findings from studies using different metrics cannot be compared (Van den Berg & Walsh 2023). Metrics used by previous studies to examine food insecurity and hunger among South African students include a single-item measure, an eight-item Household Food Security Survey Module (HFSSM), household hunger subscale (HHS) and the Household Food Insecurity Access Scale (HFIAS) (Kassier & Veldman 2013; Van den Berg & Raubenheimer 2015; Wagner, Kaneli & Masango 2021).

The Household Food Insecurity Access Scale is popular for examining food security access among diverse cultures (Coates et al. 2007). The HFIAS categorises food security as food secure, mildly, moderately and severely food insecure. Food-secure individuals have consistent access to adequate food. Mildly food-insecure individuals experience anxiety over food sufficiency. Moderately food insecure people have inferior quality, less variety and choice but still have sufficient food intake, while severely food insecure refers to irregular eating patterns and reduced food intake. This study utilised the HFIAS to investigate the food security of a cohort of South African students, especially because food insecurity among certain students was a global concern prior to COVID-19 (Cady 2016).

Prior to COVID-19, food insecurity rates of between 11% and 38% were evident among South African students from Wits University in 2019 based on HFIAS findings (Wagner et al. 2021), while rates up to 86% were found among students of the University of the Free State in 2013 by utilising a single item measure and the HFSSM (Van den Berg & Raubenheimer 2015). Food insecurity has various impacts on students, ranging from poor academic performance to nutrient deficiencies. Therefore, student food security is crucial for quality of life and academic success (Banerjee et al. 2021). South African higher education institutions assisted students during COVID-19 with resources such as support services and mobile data (Mungadze 2020). Limited evidence supports the success of maintaining students’ health and well-being. Moreover, because students were studying remotely during COVID-19, their awareness and access to support services could be affected. The objectives of this study were to assess student’s food security during COVID-19, to examine dietary intake and to explore awareness and utilisation of institutional support services.

## Research methods and design

Focus group discussions and questionnaires were used as methodologies in this concurrent, exploratory mixed methods study (Tashakkori & Teddlie 2010). Focus group discussions provided detailed, qualitative (mainly explorative) data to build on the quantitative questionnaire data (mainly confirmative). The study population comprised Health Sciences students at a university of technology in South Africa in 2021.

### Data collection

#### Questionnaire sampling

No sampling was performed for the questionnaire, as the entire population of Central University of Technology students was eligible to participate and had equal access and opportunity to participate. The researcher was not aware of any barriers to access as students utilised the same online platform used for teaching and learning. Moreover, students were encouraged to complete the questionnaire while on campus to utilise the available campus wireless fidelity (Wi-Fi), which was stable and free to registered students. The inclusion criteria of the target population who were recruited as participants were as follows: participants from the four Health Sciences training courses listed as health workers, as well as participants in their final years of study during 2021, as these students are more familiar with their future industry. Students were invited to participate via the institution’s online teaching platform, and invitations included a link to the electronic questionnaire. All participants had to provide informed consent to gain access to the questionnaire.

#### Questionnaire

The questionnaire included sections on sociodemographic information, food security status (based on the HFIAS), dietary intake and nutrition-related support services at the institution. The original HFIAS measures the access components of household food insecurity and consists of nine questions in three different domains. This HFIAS was minimally adapted by changing the period being investigated to a longer period, thus investigating the COVID-19 period instead of 30 days (as in the original scale). Furthermore, the nine questions from the three domains were all included, as in the original and previously validated HFIAS (Kassier & Veldman 2013; Van den Berg & Raubenheimer 2015; Wagner et al. 2021). Dietary intake was investigated using questions from the SA Demographic and Health Survey (SADHS) that has previously been applied in other studies in South Africa (Nwosu et al., 2022) and consists of a 30-item food frequency questionnaire. The food types of thirty items were grouped together (with the assistance of a dietician) to result in fewer (16) items while still including all food types included in the original SADHS tool. Participants had to indicate a change in intake frequency for each item, if any. The last questionnaire section investigated the awareness and utilisation of the available support services at the specific institution during the pandemic to determine whether the services influenced students’ food security and dietary intake. Additionally, this section assessed whether additional support services were needed. Therefore, data from the section provided direction for institution to support students during future ICE events.

A pilot study was conducted prior to data collection to ensure quality and to determine feasibility of the questionnaire. Additionally, the validity of the questionnaire as a quantitative data collection tool was addressed. The validity was confirmed by using existing data collection tools, as well as performing a reliability test on the Likert scales with Cronbach alpha values. According to the Cronbach alpha values, all the questionnaire sections showed good internal consistency and reliability.

#### Focus group discussion sampling

Systematic random sampling was utilised to select participants for focus group discussions. The inclusion criteria for focus group participants were the same as the criteria for a questionnaire target population. Although eight participants were recruited per focus group session, two to three participants were present to participate in the discussions.

#### Focus group discussions

Six semi-structured focus group discussions were held guided by a discussion schedule. The schedule included probes on food access and dietary intake during COVID-19. Additionally, awareness and utilisation of available support services were discussed. The focus group discussions were facilitated by an experienced facilitator from the institution being investigated. Although the facilitator was familiar with some participants, confidentiality to protect participant identity was prioritised. Field notes, recordings and transcripts which captured discussions were moderated by an external moderator.

A pilot study was conducted prior to focus group discussions to ensure quality, as well as to determine whether the discussion schedule and probes were able to effectively guide the discussion. Furthermore, the trustworthiness of the focus group discussions as a qualitative data collection tool was addressed. Trustworthiness aspects included credibility, confirmability, dependability and transferability (Noble & Smith 2015). Credibility and confirmability of the study were addressed by sufficient involvement of the researcher, methodological triangulation (both questionnaires and focus group discussions), data triangulation (field notes, recordings and transcripts), peer debriefing and scrutiny (by a moderator, supervisors and statistician) and member checking with participants during the discussion to confirm the accuracy of data. Additionally, the peer debriefing and scrutiny, member checking and triangulation increased the dependability of the focus group findings. Lastly, transferability was improved by detailed descriptions of methods, context and circumstances of the research setting.

### Analysis of data

Data analysis was performed according to Vogl’s (2018) four-step process of mixed analysis. This analysis served a dual purpose of complementarity and expansion, where the data from the focus groups provided elaborative detail and contextualised questionnaire data. Initially, a basic parallel analysis was implemented where data from questionnaires and focus group discussions were analysed in isolation.

Questionnaire data was evaluated by means of descriptive statistics utilising SPSS software and included: frequencies and percentages (for categorical data), as well as means and standard deviations (for symmetrical numerical variables). Additionally, non-parametric statistics and correlation analysis were performed to investigate correlations. The parametrical statistics were preceded by normality and reliability assessments of variables. The Shapiro-Wilk test was used for the normality assessment and the Cronbach alpha to assess the reliability (Laerd Statistics 2019). According to reliability assessments, all variables scored higher than 0.804, indicating acceptable levels of reliability. Frequencies and percentages were interpreted against the scales of the existing data collection tools.

Simultaneously, focus group discussions were prioritised, categorised and summarised prior to thematic analysis. The thematic analysis aimed to identify, analyse and report themes. The analysis was conducted according to the process described by Maguire and Delahunt (2017), and ATLAS.ti9 software facilitated the manual thematic analysis. Context-specific themes emerged, and thematic saturation was reached already with focus group two.

Following this first step of mixed analysis, findings from the isolated, parallel analysis were integrated and consolidated into one set of data. The final analysis was performed on this consolidated data by means of descriptive and interferential statistics. This analysis was based on explorative data analysis that included more graphical analysis, association and significance testing.

### Ethical considerations and statement of reflexivity

Ethical clearance to conduct this study was obtained from the University of the Free State, Health Sciences Research Ethics Committee (reference no.: UFS-HSD2021/0762/21).

The researcher considered four dimensions of reflexive processes: personal, interpersonal, methodological and contextual as described by Olmos-Vega et al. (2022). Reflexive writing and collaboration were methods integrated in the study to control reflexivity of the researcher and only a few examples are highlighted. Personal reflexivity: as an academic at the higher education institution the research problem was identified from personal experiences and observations, while an external research panel reviewed and verified the relevance of the study prior to conducting it. Interpersonal reflexivity: Participants might have been familiar with the focus group facilitator as an employee at the institution; therefore field notes were externally moderated against discussion recordings to identify interpersonal dynamics that could have influenced responses. Methodological reflexivity: A mixed-method research approach was not the only option available. However, the researcher discussed the pros and cons of the approach with supervisors, and a collaborative decision was made that it was the most suitable approach for the current study. Contextual reflexivity: The researcher was familiar with the higher education context; however, during COVID-19, unfamiliar circumstances resulted which were assumed to have had an impact on higher education.

## Results

### Sociodemographic information

A total of 289 students received and accessed the questionnaires, while only 159 students agreed to participate. Therefore, a response rate of 55% was achieved. The 159 questionnaires included 100 complete and 59 incomplete questionnaires. The majority (49) of the incomplete questionnaires were usable.

Moreover, 17 students participated in the six focus groups. Most questionnaire participants (*n* = 149) were females (82%), while only 18% were males. Focus group participants (*n* = 17) were all females. The participants were between 20 and 35 years old, of which 88% were in their early twenties (< 25 years). Questionnaire participants (*n* = 149) resided either at home or in residences. More than half of the participants were residing at home, either with caretakers (36%) who assisted with purchasing groceries and cooking or without caretakers (33%). The rest spent most of the pandemic in residences where meals were not served (30%), while few residence students received meals (1%). In addition to the institution requesting students to vacate residences if at all possible, focus group participants indicated that a lack of income was a reason for students returning home, as parents could not afford to support two households. Seventy-six percent of students resided in urban areas (town/city) and 24% in rural areas during the COVID-19 pandemic. Despite students from rural areas indicating that areas of residence had limited grocery stores and stock in those stores, it was also highlighted during focus groups that certain students from these areas had the privilege to plant and produce their own fresh vegetables. However, this was on a small scale and delivered limited, insufficient produce.

### Food security

Data from the HFIAS questionnaire described characteristics of, and changes in participants’ food insecurity (access). These indicators provided an overview of Household Food Insecurity Access-related Conditions, Household Food Insecurity Access-related Domains, HFIAS Score and Prevalence. A total of 141 participants completed all the questions in this section (Table 1).

**TABLE 1:** Students’ food insecurity access-related conditions and domains including frequency (*N* = 141).

Household Food Insecurity Access-related Domains	Questions enquiring how often students experienced the following during COVID-19	Never (not even once) %	Rarely (1–2 days per month) %	Sometimes (> 2 days but < 10 days per month) %	Often (> 10 days per month) %
Category 1: Anxiety and uncertainty about food supply	Worry that you would not have enough food	30	28	31	11
Category 2: Insufficient quality (includes variety and preferences of food type)	Unable to eat the kinds of foods you preferred because of a lack of resources	31	23	29	16
	Ate just a few kinds of food day after day because of a lack of resources	32	29	26	12
	Ate food that you preferred not to eat because of a lack of resources to obtain other types of food	37	31	20	12
Category 3: Insufficient food intake and its physical consequences	Ate a smaller meal than you felt you needed because there was not enough food	46	28	20	6
	Ate fewer meals in a day because there was not enough food	50	22	21	7
	Went to sleep hungry because there was not enough food	71	21	6	2
	Went a day without eating anything because there was not enough food	80	14	4	2
	No food at all in your household because there were no resources to get more	73	21	5	1

COVID-19, coronavirus disease 2019.

#### Household food insecurity access-related conditions

This selection of findings reflects specific, disaggregated information about the behaviours and perceptions of the participants. Table 1 presents each indicator separately to illustrate the frequency of experiencing the conditions. While 70% of students worried about not having enough food and 27% of students experienced having no food in their homes, both these groups are classified as food insecure despite the varying levels of severity. A noteworthy observation is that 29% of participants went to bed hungry some nights, whereas 50% had to cut down on their daily number of meals.

It should be noted that the students who had food could not necessarily eat what they preferred but had to consume what was available, as per results from questions 3 and 4. Similarly, focus groups reported that although students may have had adequate food, it was not necessarily the food they used or preferred to consume. A probable reason may be the lack of variety available in retail outlets, as well as limited financial resources and area of residence. The findings indicate that hunger could not be prevented, and certain students experienced food insecurity during COVID-19, at various frequencies, despite the government and institutions’ available assistance.

#### Household food insecurity access-related domains

The HFIAS questions relate to three different domains of food insecurity and findings were clustered into these domains to provide insight into each domain. Table 1 presents the clustered findings with the percentage of participants that responded affirmatively to each question, regardless of the frequency of the experience. Anxiety and uncertainty about food supply often occurred among students (category 1), with 70% of students worrying about their food supply during COVID-19. Insufficient quality (category 2) of food was experienced by 67% of students. While this indicates food insecurity without definite hunger, the impact of inadequate quality of food remains a concern. Category 3, insufficient food intake and its physical consequences, includes more serious food insecurity factors such as hunger. In comparison to category 2, this dimension’s percentage of occurrence was lower and 36% of students experienced a lack of food intake during COVID-19. The three domains indicate a ranking of severity with category 1 being mild and category 3 severe. Therefore, although a higher score in the mild category 1 and a lower score in the severe category 3 existed, the pattern may suggest that students with milder food insecurity might have experienced worse levels of food insecurity should the ICE situation have persisted.

#### Household food insecurity access scale score and prevalence

Although the findings illustrated per domain provided some information on the level of severity, the following findings provided more details on the severity levels of food insecurity. The HFIAS’ nine questions provided a continuous measure of the degree of food insecurity (access) with a maximum score of 27 (when the participant’s response was ‘often’ to all nine questions, coded with a response code of 3, and the minimum score is 0 indicating no food insecurity). Thus, the higher the score, the more food insecurity the student experienced.

Findings indicated that 16% of participants were food secure and thus had consistent access to adequate food. More than half (54%) of the students had a food insecurity score of 1–10 indicating mild food insecurity including anxiety over food sufficiency despite sufficient intake. Twenty-eight per cent of students scored between 11 and 20, reflecting moderate food insecurity with inferior quality, less variety and choice of food. The highest food insecurity scores (21–27) were obtained by 2% of the students who had severe food insecurity with intermittent eating patterns and reduced food intake. Financial constraints were the main reason for food insecurity. Other reasons included: limited time to prepare food and the inability to travel to food suppliers.

#### Overview of focus group findings on food security

**FIGURE 1:**
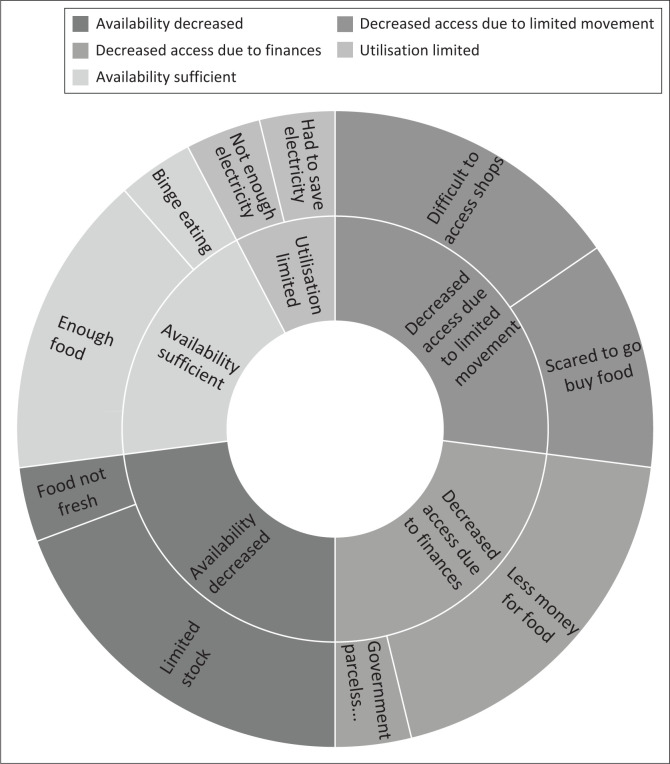
Food security-related themes and codes (*N* = 17).

The focus group discussion probed food security issues, and Figure 1 illustrates the common denominators using emerging themes and codes. The four themes related to food insecurity included availability decreased, decreased access because of limited movement, decreased access because of finances and limited utilisation. Emerging codes were aligned with the results from the questionnaire’s section on food security. The following codes emerged as reasons for food insecurity, in addition to reasons obtained from the questionnaires: available food was not fresh, access was limited because of fear of travelling to shops, support from government was not sufficient, utilisation was affected by limited electricity and attempting to save electricity because of finances. Contradicting the reason for food insecurity because of poor appetite; some focus group participants indicated that their appetite increased, and many practised binge eating habits to cope with anxiety.

### Dietary intake

**FIGURE 2:**
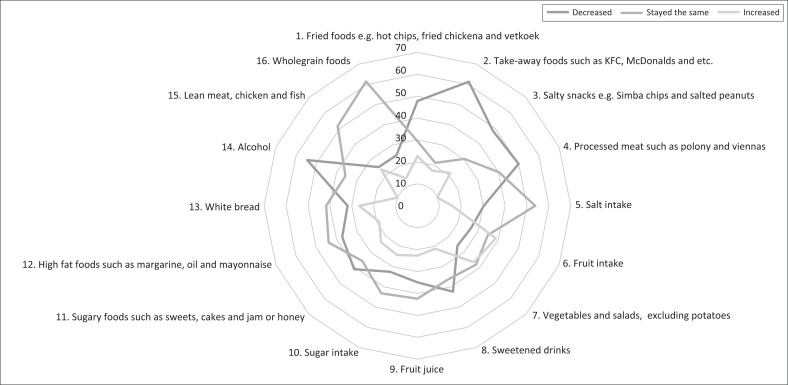
Food frequency intake change of students during the COVID-19 pandemic (*N* = 132).

Findings from this section of the questionnaire provide insight into the changes in students’ dietary intake regarding specific types of food. Figure 2 illustrates that most of the students’ dietary intake (*N* = 132) decreased or was maintained, with limited types of food intake that increased during COVID-19. Therefore, this section’s dietary intake results aligned with the results from the previously discussed food insecurity section. More than half of the students indicated a decreased intake of takeaway foods (61%) and alcohol (55%). Other food types that were indicated to have a decreased intake during COVID-19 were fried foods (48%), salty snacks (49%), processed meat (50%), sweetened drinks (42%) and sugary foods (41%). While this points to a healthier diet, students from focus groups indicated that they often resorted to comfort eating when they had such snacks available.

Another noteworthy finding was that participants indicated an increase or maintenance in fruit and vegetable intake. In the fruit intake category, 35% of students stated their intake stayed the same and 39% indicated an increased intake. Moreover, in the vegetable and salad category, 38% of students indicated a similar intake than before and 36% indicated an increased intake during COVID-19. These findings indicate that despite focus group students’ statements on the limited availability of fresh produce, less than a third of students experienced a decreased intake of fruit and vegetables. Another reason for the limited decrease in intake may be subsistence farming, as focus group participants stated that they often relied on their own fresh produce, although these were limited. Like fruit and vegetable intake, more than half of the students indicated that their meat intake was maintained. This finding may be justified by the same reasoning as for the intake of fruits and vegetables. Food types in the starch category stayed the same for most students. White bread intake stayed the same for 42% of students, and wholegrain foods stayed the same for 61% of students. In contrast to the findings on starch, focus group participants suggested that their starchy food intake increased during COVID-19, specifically instant noodles and mealie meal, which were more affordable.

The Shapiro-Wilk normality assessment of the food security variable, resulting in a *p*-value < 0.05, indicated a significant deviation from a normal distribution. Therefore, the non-parametric equivalent tests were conducted, which do not assume normality in the data. The Mann-Whitney non-parametric test compared the differences between two independent home residence groups: urban and rural. The aim was to determine whether the home residence was associated with food security.

The food security total score showed a statistically significant difference in the mean rank scores of students living in urban residences compared to students living in rural residences (Z = –2.318, *p* = 0.020). As is evident from Table 2, the mean rank scores of the food security variable of students living in rural residences (83.87) were much higher than students living in urban residences (65.51). This result indicates that the home residence of students had an influence on their food security score, with students living in rural communities having higher levels of food insecurity. A likely reason for the higher food insecurity of students from rural areas was mentioned during the focus group discussion of the current study:

‘I live in a in a very small town, so there are no like shops or limited, the shops are limited … we went to buy food and then when you get to the shop … we had to take whatever that was there ….’ (Focus group 1, Female, Somatology course)

**TABLE 2:** Mean rank scores according to home residence categories.

Variable	Home residency	*n*	Mean rank	Sum of ranks
**Food security total score**	Urban (town/city)	105	65.51	6878.50
	Rural settlement (farm/location)	34	83.87	2851.50

**Total**	**-**	**139**	**-**	**-**

The results of the Mann-Whitney *U* comparison showed no statistically significant difference between the gender categories. Therefore, students’ gender did not influence their food insecurity.

### Support services

The results of the support service section indicated that 82% of students were not aware that any support services to assist students with health-related issues were available at the specific institution. Moreover, only 1% of students indicated that they were using the institutional food programme. Although 18% of students indicated they were aware of support services, the many irrelevant responses from the open-ended question that investigated which services they were aware of indicated that not all 18% were aware of the actual services. This might indicate that some students lacked knowledge of what support service entailed and that a need existed for information on the purpose of services. Focus group discussions yielded similar responses regarding the available support services at the specific institution during the pandemic. Limited students were aware of available support specifically related to food security. Additionally, concerns were raised by focus group participants regarding the accessibility of the available support services, such as food parcels, where students had to complete a complicated process of administration to qualify, as well as statements from students in need who were not confident to approach support services.

Participating students from focus group discussions requested assistance and additional services such as dietary guidelines/healthy eating programmes. These discussions included requests to be mindful of students’ circumstances, such as lack of funds, to purchase a variety of foods or to obtain resources needed to prepare the food.

## Discussion

### Sociodemographic information

Although most of the participants were female, the study was not reliant on gender ratio, and the skewed gender ratio did not affect the findings. Most students lived at home during COVID-19, which was expected as students had to vacate their campus and private residences because of COVID-19 regulations (Hendricks & Chirume 2020). However, students did not necessarily have the support of parents/caretakers at their homes. Only a third of the students had support from caretakers who could assist with household tasks such as cooking and a theme arose from the focus groups about students managing the households and often being the breadwinners of their households. A total of 33% of the participants had to manage their own households. These sociodemographic findings could play a role in the results about food security, as caretakers may assist with students’ access to and availability of food. Similarly, findings from a South African study showed that child-headed families struggled more with poverty and poor nutrition (Makuya 2022).

### Food security

While students from Central University of Technology received some degree of assistance from the institution (e.g. food parcels and mobile data) (Mungadze 2020), questionnaire findings indicated that about 30% of the students maintained food security access, and focus group discussions highlighted themes indicating concerns regarding availability and utilisation. These findings concurred with evidence from other studies before and during COVID-19 (UNICEF 2021).

Food insecurity among students was not a new tendency at the international or national level and had been a concern before the pandemic (Kassier & Veldman 2013; Van den Berg & Raubenheimer 2015). International estimates before COVID-19 suggested that half of the United States’ students experienced some degree of food insecurity according to four studies from 2009 to 2016 using the six-item survey module (Broton & Goldrick-Rab 2018). Similarly, a study in 2019 utilising the 10-item US Household Food Security Survey Module found that 80% of students from two universities in Nigeria were food insecure (Ukegbu et al. 2019).

Nationally, four studies from 2012 to 2019 (before the pandemic) reported food insecurity among South African students. In 2012, Kassier and Veldman (2013) assessed students at the University of KwaZulu Natal using the HFIAS and found 12.5% of students to be food insecure. A study at the University of the Free State in 2013 identified various levels of food insecurity among 64.5% of students using a single-item measure and 84.6% of students using the eight-item Household Food Security Survey Module (Van den Berg & Raubenheimer 2015). Similarly, a study conducted at Witwatersrand University in 2019 reported food insecurity among students before COVID-19. This study found various levels of food insecurity among 73% of students with the HFIAS and degrees of hunger among 13% of students using the HHS (Wagner et al. 2021). Therefore, considering the findings of the mentioned studies, it was expected that the COVID-19 pandemic and regulations would have increased prevalence of food insecurity among students.

This expectation was confirmed by the current study and other studies among the general population. UNICEF (2021) indicated a significant global upward trend in food insecurity between 2019 and 2020. Furthermore, Van der Berg, Patel and Bridgman (2022) indicated the rates of South African hunger doubled in the initial months of COVID-19. Similarly, Statistics SA indicated increased food insecurity among South Africans from 17.3% in 2019 to 23.6% in 2020 (STATSSA 2022). These increased rates were strongly related to the availability of money to buy food (Van der Berg et al. 2022) and the unemployment rate (STATSSA 2022). This concurred with this study’s findings.

Additionally, findings from the food security questionnaire section indicated that more students experienced food insecurity within the first two domains: ‘anxiety and uncertainty about food supply’ and ‘insufficient quality’. The pattern within the findings was aligned with the consistent pattern that Radimer identified with qualitative interviews and a 30-item hunger survey conducted among women. The pattern was as follows: the lived experience of food insecurity was initially characterised by anxiety and worry about enough food. Then, as conditions worsened, it resulted in less stored food in the home, followed by worsening quality and diversity of the diet, decreased quantity of food per meal and, finally, being forced to skip meals and feel hungry (Radimer et al. 1992). This might be the case even after the pandemic ended, as the negative impact thereof on other factors such as the economy did not improve with immediate effect (Van der Berg et al. 2022). These findings may assist institutions with decisions on student food insecurity-related priorities when considering interventions and policies.

Moreover, compelling evidence suggests that food insecurity among students is associated with decreased academic performance, which in turn may hinder the graduation rate and economic growth of the country (Goldrick-Rab et al. 2018; Raskind, Haardorfer and Berg 2019; Sabi et al. 2019). The reverse was also evident from studies showing a direct link between good diet quality and higher academic achievement (Raskind et al. 2019). Furthermore, the effects of food insecurity on psychosocial health impede students’ academic performance and contribute to high exhaustion levels (Van den Berg & Raubenheimer 2015). Therefore, this study’s findings and existing literature indicate that COVID-19 increased the urgency of policies and interventions to alleviate food insecurity and possibly maintain or increase students’ success rate.

The questionnaire findings showed no association existed between food security and gender. The skewed gender ratio could have impacted the findings on gender association. However, an association existed between food security and the two home residence categories. This association with home residence indicated that students living in rural communities had higher levels of food insecurity. A likely reason might be financial insufficiency, as households from rural areas tend to have lower incomes (Mathebula et al. 2017). This probable reason supports the study by Diamond, Stebleton and Del Mas (2020) that found a positive association between food insecurity (short and long-term) and students who rely on study grants and/or are from lower-income households. Because COVID-19 influenced the economy, which has still not recovered (Chitiga-Mabugu et al. 2021), the possibility of financial insufficiency among rural households is a concern that needs urgent attention. Another probable reason for higher food insecurity among students from rural settlements could be the lack of access to food, as mentioned during the focus groups of the current study. While there might be various reasons for the positive association between rural residence and food insecurity, higher education institutions should prioritise investigating the matter. Moreover, this association could be useful to identify students at risk of food insecurity upon registration, and institutions can establish mitigation measures to ensure food security and academic success.

### Dietary intake

Questionnaire findings indicated that the frequency of intake of most foods was either maintained or decreased. Some of these rates were expected, as South African COVID-19 regulations placed restrictions on the fast food and alcohol industries (South African Government 2020) despite the nutrition transition of South Africa as described by Ronquest-Ross, Vink and Sigge (2015). Another probable reason was that many of these foods were considered luxuries and necessities were prioritised during the pandemic (Van der Berg et al. 2022). This probability reflects the influence of economic factors as mentioned in the three prevalent conceptual models of food choice (EUFIC 2006).

Comfort or binge eating was a theme from the focus groups and was linked to the anxiety and depression caused by the pandemic circumstances. Literature indicates that a stressful event causes anxiety and depression, which in turn, result in unhealthy eating patterns, including overeating, binge eating and eating disorders (Ukegbu et al. 2019). Moreover, this becomes an alarming cycle of food insecurity (Shi, Davies & Allman-Farinelli 2021). Focus groups included a theme indicating lower intake of fruits and vegetables in contrast to the questionnaire findings. During COVID-19, the regulations made provision for farmers to continue production, and as a result, some fruits and vegetables were still available (Fruit SA 2021). However, import and export regulations during COVID-19 resulted in a shortage of certain fresh produce (Fruit SA 2021). This evidence justified the claim made by focus group participants.

Another theme from focus groups contradicting findings from questionnaires was the increase of starchy food, especially foods that needed fewer resources to prepare, for example, instant noodles and bread. While the contradiction is difficult to explain, this food choice from focus group findings relates to the physical determinant category mentioned in the EUFIC food choice model (EUFIC 2006). Moreover, in South Africa, it is known that a large part of the population relies on starchy foods, such as bread and mealie meal, as the main part of their diet (Van Heerden 2013). Therefore, the findings should be considered in the light of the South African context, though the intake of starchy food stayed the same. Despite basic food intake guidelines suggesting starch should be the basis of all meals, students’ intake of starch was not necessarily in line with dietary recommendations (WHO 2003) and should be viewed in relation to the intake of the other major food groups such as fruits, vegetables and meat.

According to conflicting results from surveys and focus groups, some students’ diets were healthier, and others were less healthy during the pandemic. The students’ access to healthier meals in their off-campus homes may contribute to their improved diets. On the contrary, when students’ homes were food insecure or lacked caretakers to help with food access, students may have reverted to poor dietary habits.

### Support services

Findings indicated most students were not aware of the available support services at the relevant institution. In addition to the need that existed for institutions to increase awareness of available services, a need for additional or expansion of services also became apparent – although the possibility existed that capacity and related budgetary issues, as described by Moyo and McKenna (2021), may make it difficult for institutions to meet students’ needs. As is evident from this and other studies, the pandemic has led to an increase in food insecurity among students, of which many were already vulnerable before the pandemic (Ukegbu et al. 2019; Van den Berg & Raubenheimer 2015). Although some universities had already implemented food relief programmes and other support, a growing need existed for more comprehensive and sustained efforts.

The COVID-19 circumstances resulted in a sudden increased need, and literature predicted that certain effects, such as poverty, would not disappear soon after the pandemic ended (UNICEF 2021). Moreover, international concerns such as climate change, conflict and population increase further impact the availability and/or access to nutritious food. Therefore, it is essential that institutions either implement or review food relief programmes to meet the increased needs and be prepared for similar future events.

Because of challenges such as resilience and cost constraints (Barlett 2011), there is not a single food relief programme structure/model to suit all higher education institutions. However, Lowe (2021) suggested that improving higher education food relief programme’s cost-effectiveness and sustainability should be a matter of urgency. Strategies mentioned to improve such nutrient density included supplementation, fortification, biofortification and diet diversification. Furthermore, institutions should consider the socio-economic circumstances and specific needs of their diverse student populations in relation to the available resources in order to develop a sustainable food relief programme that will adequately support students. Additionally, as evident from the current study, institutions should also take cognisance of students who might not be confident to reach out to food relief programmes (Sabi et al. 2019). Without support to improve students’ food security, many students might be forced to discontinue their studies, further exacerbating the social and economic challenges faced by the country (Van den Berg & Raubenheimer 2015).

## Conclusion

While findings from this study indicated food insecurity, hunger and decreased dietary intake during COVID-19, there were no related data on this population before COVID-19 to perform a comparative analysis. However, evidence from similar studies suggested that the current study’s findings could have been prevalent before COVID-19. Therefore, it is recommended that future studies investigate students’ food security outside of the pandemic period. Despite significant investment in food security and other socio-economic areas of students by the government and higher education institutions, food insecurity and hunger have resulted from COVID-19 circumstances. It is also worth noting that the available food relief programmes and institutional support services were not as effective because of reasons such as lack of awareness, difficulty in access and pride discouraging utilisation.

Given the prevalence of food insecurity and hunger before COVID-19 and the extended nature of the pandemic, including slow economic recovery, support for vulnerable students became an urgent priority. It is assumed that the reflected levels of food insecurity and hunger among students would remain high because of factors such as the removal of emergency relief grants and food support and the slow rate of economic recovery. Therefore, the need for higher education and government intervention is essential, given the dire consequences of enduring hunger on students’ quality of life and academic performance.
